# Supplementary material for: Pathological Evidence for Residual SARS-CoV-2 in the Micrometastatic Niche of a Patient with Ovarian Cancer
Source: Curr Issues Mol Biol. 2022 Nov 26;44(12):5879–89. doi: 10.3390/cimb44120400 (PMC9776863; doi:10.3390/cimb44120400)
Supplement: Supplementary file 1 [file cimb-44-00400-s001.zip › Enago-Certificate-TABUWJ-57..pdf]

enago
Author First, Quality First
www.enago.jp

ulatus
localize to globalize
www.ulatus.jp

voxtab
we are all ears
www.voxtab.jp

CERTIFICATE OF EDITING

This is to certify that the paper titled NA commissioned to us by Takuma Hayashi has been edited for English language, grammar, punctuation, and spelling by Enago, the editing brand of Crimson Interactive Pvt. Ltd under Normal Editing B2C.

✔ **ISO 17100:2015**
Translation Service Providers

✔ **ISO 27001:2013**
Information Security Management System

✔ **ISO 9001:2015**
Quality Management System

Issued by:
Enago, Crimson Interactive Pvt. Ltd.
1001, Techniplex - II, S. V. Road,
Goregaon (W), Mumbai 400062, India.
Phone: 03-5050-5374
Fax: 03-4496-4934

Disclaimer : The intent of the author's message has been preserved during the editing process. The author is free to accept or reject our changes in the document after reviewing our edits. This certificate has been awarded at the time of sharing the final edited version (full file or sections of the file) with the author. Enago does not bear any responsibility for any alterations done by the author to the edited document post 21 10月 2022.

**Japan**	www.enago.jp, www.ulatus.jp, www.voxtab.jp
**Taiwan**	www.enago.tw, www.ulatus.tw
**China**	www.enago.cn, www.ulatus.cn
**Brazil**	www.enago.com.br, www.ulatus.com.br
**Germany**	www.enago.de
**Russia**	www.enago.ru
**Arabic**	www.enago.ae
**Turkey**	www.enago.com.tr
**S. Korea**	www.enago.co.kr
**Global**	www.enago.com, www.ulatus.com, www.voxtab.com

About Crimson:
Crimson Interactive pvt ltd is one of the world's leading academic research support services. Since 2005, we've supported over 2 million researchers in 125 countries with their publication goals.
