# Supplementary material for: Pathological Evidence for Residual SARS-CoV-2 in the Micrometastatic Niche of a Patient with Ovarian Cancer
Source: Curr Issues Mol Biol. 2022 Nov 26;44(12):5879–89. doi: 10.3390/cimb44120400 (PMC9776863; doi:10.3390/cimb44120400)
Supplement: Supplementary file 1 [file cimb-44-00400-s001.zip › f.Supplementary material.10.24.2022_INQ-6154161822_TABUWJ-53.pdf]

Supplementary Information

Supplementary Materials for

Pathological evidence for residual SARS-CoV-2 in the micrometastatic niche of a patient with ovarian cancer

Takuma Hayashi, Kenji Sano, Nobuo Yaegashi, Ikuo Konishi

1. History of the clinical situation of a cancer patient with COVID-19

A 47-year-old woman sought a consult at the outpatient clinic with a 3 day history of fever, cough, and shortness of breath. With this, the patient underwent a COVID-19 PCR test (Supplementary Table S1). Moreover, relevant in her medical history is her regular visits with a gynecologist at a nearby general hospital for ovarian tumors.

Information provided to the doctor in charge of the primary medical facility

1. Urgent surgery may be necessary. However, in our medical facility, surgery may be performed later in the middle of the night. If the patient is complaining of significant pain, the attending physician should consult with another hospital where surgery can be performed early.
2. Since the patient is COVID-19 positive, a contact procedure with the health center is required during patient transport to our institution, which is the designated national hospital for accepting highly acute phase/tertiary critical care/COVID-19 severely ill patients.

(Supplementary Table S1)

A nasopharyngeal swab from the patient was collected by our medical staff for rapid RT-PCR testing, which rendered positive results for COVID-19. A chest X-ray was performed, which showed diffuse bilateral radio-opacities, that is, a frosted glassy shadow.

On examination, the patient was afebrile, pulse rate was 79 beats per minute, blood pressure was 122/81 mmHg, respiratory rate was 16 breaths per minute, and oxygen saturation on room air was 93%. The patient's random blood sugar was 110 mg/dL. The patient was admitted to the high care unit ward with an isolation facility and started on oxygen support to maintain a saturation of ≥95%. Initial investigations showed a neutrophil count (4.7), elevated C-reactive protein (0.47 mg/dL), lactate dehydrogenase (178 U/L), low Hgb level (11.5 g/dL), low serum Fe level (21 µg/dL), and decreased white blood cell count (5700/µL).

Three days after the standard surgical treatment for ovarian tumors (including ovarian tumor torsion), the patient was stable and was transferred to a nearby general medical facility. The attending doctors of the nearby general medical facility were clinically treating the patient for COVID-19 and conducting surgical follow-up.

2. Materials and Methods

1. Antibodies

The list of antibodies, which were used as the first monoclonal antibody or secondary antibody in our immunohistochemistry research experiments, is shown below.

Antibodies

Antibody	Company	Catalog No.	Clonal (Clone)	Specificity	Dilution
S100A4	Abcam	ab124805	Rabbit monoclonal EPR2761(2)	Human	1:200 (IHC)
CD90	Abcam	ab133350	Rabbit monoclonal EPR3133	Human	1:200 (IHC)
ACE2	ORIGENE	CF803844	Mouse monoclonal 4C5	Human	1:150 (IHC)
RBD of spike	GeneTex	GNT-9366-04	Mouse monoclonal 1A9 (GNT936604)	SARS-CoV-2	1:200 (IHC)
Antimouse IgG Alexa Fluor® 488	Invitrogen	A32723	Goat/IgG	Mouse IgG	1:100
Antirabbit IgG Alexa Fluor® 546	Invitrogen	A-11035	Goat/IgG	Rabbit IgG	1:200

2. Immunohistochemistry (IHC)

IHC staining for CD90 (Thy1), S100A4, ACE2, and RBD of spike glycoprotein of SARS-CoV-2 was performed on tissue sections of HG-SOC. Antibodies for CD90 (Thy1) (ab133350) and S100A4 (ab124805) were purchased from Abcam Inc. (Cambridge, UK). RBD of the spike glycoprotein was purchased from GeneTex Inc. (Irvine, CA, USA). DAPI mounting medium was purchased from VECTOR LABORATORIES, Inc. (Burlingame, CA). IHC was performed using normal methods with the primary antibody and second antibody conjugated with immunofluorescence as described previously. Briefly, one representative 5-µm-thick tissue section was cut from a paraffin-embedded sample derived from patients with ovarian cancer. A human lung tissue array for examination with normal lung tissues[1] (Cat.# BC04002b, US Biomax Inc. Rockville, MD, USA) was also used for IHC examination. To examine the expression levels of the target molecules, we conducted immunofluorescence experiments for CD90 (Thy1) and S100A4 on paraffin-embedded tissues derived from patients with ovarian cancer. Tumor tissue sections were then incubated with the appropriate primary antibodies at 4°C overnight. We used rabbit monoclonal antibodies to S100A4 (1:200), a rabbit monoclonal antibody to CD90 (Thy1) (1:200), a mouse monoclonal antibody to human ACE2 (1:150), and RBD of spike glycoprotein of SARS-CoV-2 (1:200) as the primary antibody. The tissue sections were incubated with the mouse monoclonal antibody to RBD of SARS-CoV-2 spike glycoprotein at 4°C overnight. After being incubated with the secondary antibody, that is, the Alexa Fluor® 488-conjugated antimouse IgG antibody or Alexa Fluor® 546-conjugated antirabbit IgG antibody (1:200; Invitrogen), sections were washed and coverslipped with a mounting medium and DAPI (Vectashield; Vector Laboratories) and then visualized under a confocal microscope (Leica TCS SP8, Wetzlar, Germany) according to the manufacturer's procedure. In the photographs of the normal tissue areas, which are called connecting tissues and metastasis areas (HG-SOC), and normal ovary tissue region (cortex) as shown in Figure 2B and Supplementary Figure S4, the expression levels of each factor are calculated using fluorescent color. Human ovary tissue array for examination with normal lung tissues[1] (Cat.# BC04002b, US Biomax Inc. Rockville, MD, USA) was also used for IHC examination. Normal rabbit or mouse antiserum was used as a negative control for the primary antibody. A quantitative analysis was conducted using Image J Version 1.53m, a public domain software for image analysis (NIH ImageJ, Bethesda, MD, USA). These experiments with human tumor tissues derived from patients with HG-SOC were conducted at Shinshu University and National Hospital Organization Kyoto Medical Center in accordance with institutional guidelines (i.e., IRB approval no. M192, H31-cancer-2). The authors attended research

ethics education through the Education for Research Ethics and Integrity (APRIN e-learning program [eAPRIN]).
1. https://www.proteinatlas.org/learn/dictionary/pathology/ovarian+cancer

3. Transmission electron microscopy (TEM)
TEM was performed under a routine procedure. Tissues were fixed in 10% formaldehyde for 1 day. Briefly, specimens (approximately 1 mm × 1 mm × 1 mm in size) from each organ were fixed in 2.5% glutaraldehyde in 0.1 M phosphoric buffer (pH: 7.4) for 24 h, postfixed with 1% osmium tetroxide, dehydrated with gradient alcohol, and embedded using Eponate 12™ Kit with DMP-30 (18010, TED PELLA Inc.). Tissue blocks were then processed for electron microscopy. Ultrathin sections were cut into 100-nm-thick sections on a Leica Ultra-Microtome, collected on Formvar-coated single-slot grids, and then double-stained with uranium acetate and lead citramalic acid, separately. The sections were observed under a Hitachi HT7700 transmission electron microscope. TEM was performed by Hanaichi UltraStructure Research Institute, Co., Ltd. (Okazaki-shi, Aichi).

4. Institutional Review Board Statement and Consent to Participate
These experiments with human tumor tissues derived from patients with HG-SOC were conducted at Shinshu University and National Hospital Organization Kyoto Medical Center in accordance with institutional guidelines (i.e., IRB approval no. M192, H31-cancer-2). The authors attended research ethics education through the Education for Research Ethics and Integrity (APRIN e-learning program (eAPRIN)). The completion numbers of the authors are AP0000151756, AP0000151757, AP0000151769, and AP000351128. Consent to participate was required as this research was considered clinical research. Subjects signed an informed consent form when they were briefed on the clinical study and agreed with the content of the research.

5. Statistical Analysis
All data are expressed as the mean and standard error of the mean. Normality was verified using the Shapiro–Wilk test. For comparing two groups, the unpaired two-tailed *t* test or Mann–Whitney *U* test was used. Multiple comparisons were performed using a one-way analysis of variance with a Tukey post hoc test or a Kruskal–Wallis analysis with a post hoc Steel–Dwass or Steel test. A *p*-value of <0.05 was considered statistically significant. All statistical analyses were conducted using the JMP software (SAS Institute, Cary, NC, USA).

Supplementary Table S1. Reports published so far.

Previously report	Summary of report	Ref.
Shiozawa Y, Pedersen EA, Havens AM, Jung Y, Mishra A, Joseph J, Kim JK, Patel LR, Ying C, Ziegler AM, Pienta MJ, Song J, Wang J, Loberg RD, Krebsbach PH, Pienta KJ, Taichman RS. Human prostate cancer metastases target the hematopoietic stem cell niche to establish footholds in mouse bone marrow. ***J Clin Invest*** 2011; 121: 1298–1312.	Competition between disseminated human prostate cancer cells and hematopoietic stem cells (HSC) for the endosteal niche facilitates metastasis. Metastatic cells shed from a primary tumor compete with HSCs to engage the endosteal niche, which suggests that solid tumors use the HSC niche as metastatic niche. Human prostate cancer cells settle near stem cells in bone marrow, human prostate cancer cells promote the development of a metastatic environment that supports tumor growth.	8
Malanchi I, Santamaria-Martínez A, Susanto E, Peng H, Lehr HA, Delaloye JF, Huelsken J. Interactions between cancer stem cells and their niche govern metastatic colonization. ***Nature*** 2012; 481: 85-89.	The cancer cells drive neighbouring differentiated alveolar type 2 cells to take on a stem-cell-like fate in micrometastatic niche. Reports described cancer-associated parenchymal cells that exhibit stem cell-like features, the expression of lung progenitor markers, multi-lineage differentiation potential, and self-renewal activity.	9
Ombrato L, Nolan E, Kurelac I, Mavousian A, Bridgeman VL, Heinze I, Chakravarty P, Horswell S, Gonzalez-Gualda E, Matacchione G, Weston A, Kirkpatrick J, Husain E, Speirs V, Collinson L, Ori A, Lee JH, Malanchi I Metastatic-niche labelling reveals parenchymal cells with stem features. ***Nature*** 2019; 572: 603-608.	The cancer cells drive neighbouring differentiated alveolar type 2 cells to take on a stem-cell-like fate. Reports described cancer-associated parenchymal cells that exhibit stem cell-like features, the expression of lung progenitor markers, multi-lineage differentiation potential, and self-renewal activity.	10
Hayashi T, Sano K, Aburatani H, Yaegashi N, Konishi I. Initialization of epithelial cells by tumor cells in a metastatic microenvironment. ***Oncogene*** 2020 Mar;39(12):2638-2640.	The research group examined niches for promoting metastatic colonization using the generation of human-in-mouse ovarian cancer xenograft models in immunodeficient mice. Pathological examinations revealed the existence of S100A4-negative and CD90-positive stem-like cells in vimentin-positive normal neighboring alveolar epithelial cells.	11
Salahudeen AA, Choi SS, Rustagi A, Zhu J, van Unen V, de la O SM, Flynn RA, Margalef-Català M, Santos AJM, Ju J, Batish A, Usui T, Zheng GXY, Edwards CE, Wagar LE, Luca V, Anchang B, Nagendran M, Nguyen K, Hart DJ, Terry JM, Belgrader P, Ziraldo SB, Mikkelsen TS, Harbury PB, Glenn JS, Garcia KC, Davis MM, Baric RS, Sabatti C, Amieva MR, Blish CA, Desai TJ, Kuo CJ. Progenitor identification and SARS-CoV-2 infection in human distal lung organoids**. *Nature*** 2020; 588: 670-675.	Lung organoid scRNA-seq at time points before ciliated differentiation identified expression of the SARS-CoV-2 receptor ACE2 and processing protease TMPRSS2 mRNAs predominantly in progenitor or stem-like cells of alveolar type 2. Reseach group demonstrated functional heterogeneity among basal cells and establishes a facile in vitro organoid model of human distal lung infections, including COVID-19-associated pneumonia.	12
Hayashi T, Sano K, Konishi I. Possibility of SARS-CoV-2 infection in metastatic microenvironment of cancer. Molecules at Play in Cancer: ***Curr. Issues Mol. Biol.*** 2022, *44*(1), 233-241	In the pulmonary micrometastatic niche of patients with ovarian cancer, alveolar epithelial stem-like cells were found adjacent to the ovarian cancer. Moreover, angiotensin-converting enzyme 2, a host-side receptor for SARS-CoV-2, was expressed in these alveolar epithelial stem-like cells. Furthermore, the spike glycoprotein receptor-binding domain of SARS-CoV-2 bound to alveolar epithelial stem-like cells. The prevention of *de novo* niche formation in metastatic diseases might constitute a new strategy for the clinical treatment of COVID-19 for patients with cancer.	13

Table S2. Clinical description and test for the patient. Outline of disease (ovarian tumor and COVID-19) course and medical treatment for the patient.

Supplementary Table 2.
Outline of disease course and medical treatment for the patient.

	5/16	5/17	5/18	5/19	5/20	5/21	5/22
	Medical treatment at home		hospitalization				Discharge
Event	COVID-19						
			High grade serous ovarian cancer + STIC				
Symptoms	Cough, sputum, sore throat			When falling asleep, a decrease in blood oxygen concentration was observed.		normal body temperature (36.7~37.1℃), normal oxygen saturation (98.0~99.0%)	
			Severe lower abdominal pain				
Check	PCR test Virus RNA (+)	X-ray image inflammation					
			CT images Ovarian mass				
Treatment		Administration of analgesics Sosegon ➡ Acelio		Take acetaminophen when you feel pain			
				Urgent surgical treatment			The doctors in charge of a nearby general medical facility is clinical treating the patient for COVID-19 and following up after the operation.
				Oxygen Supplementation			

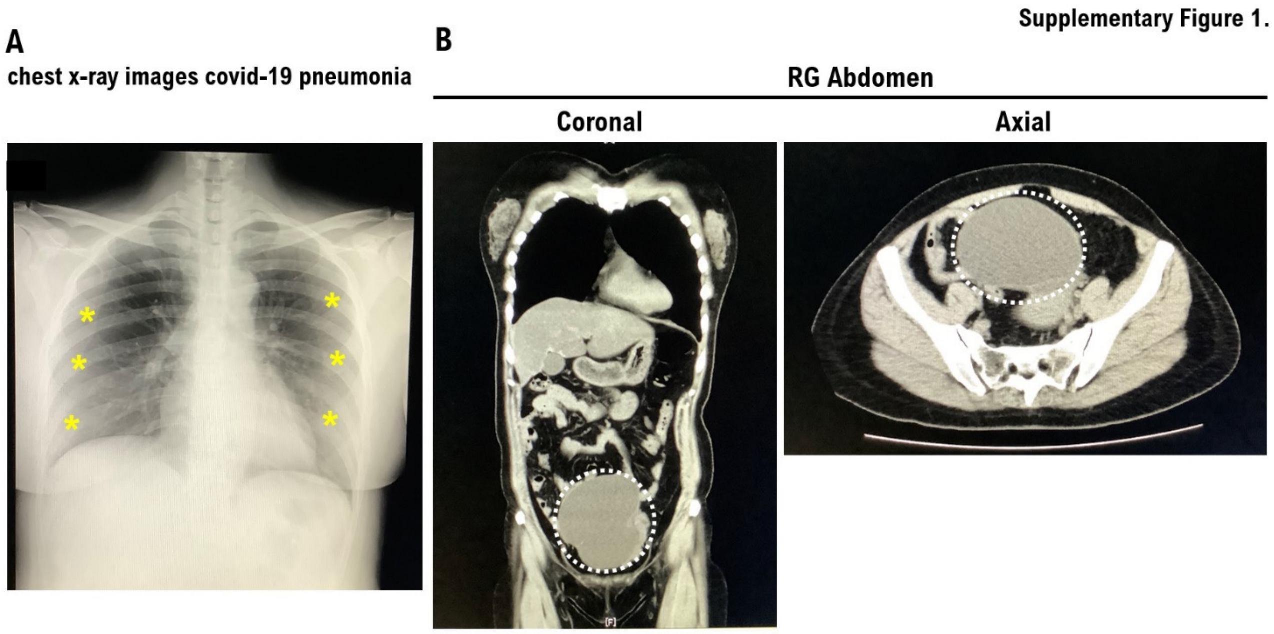

Figure S1. Suspicion of left ovarian tumor volvulus in a patient with COVID-19.
A. Chest X-ray showing bilateral radio-opacities suspicious for COVID-19 pneumonia. The chest X-ray showed bilateral radio-opacities, which are indicated by yellow star marks. **B.** The white dotted circle indicates the ovarian tumor. Enhanced abdominal CT scans (left panel, coronal plane; right panel, axial plane). These imaging findings suggested the possibility of left ovarian tumor volvulus.

Supplementary Figure 2.

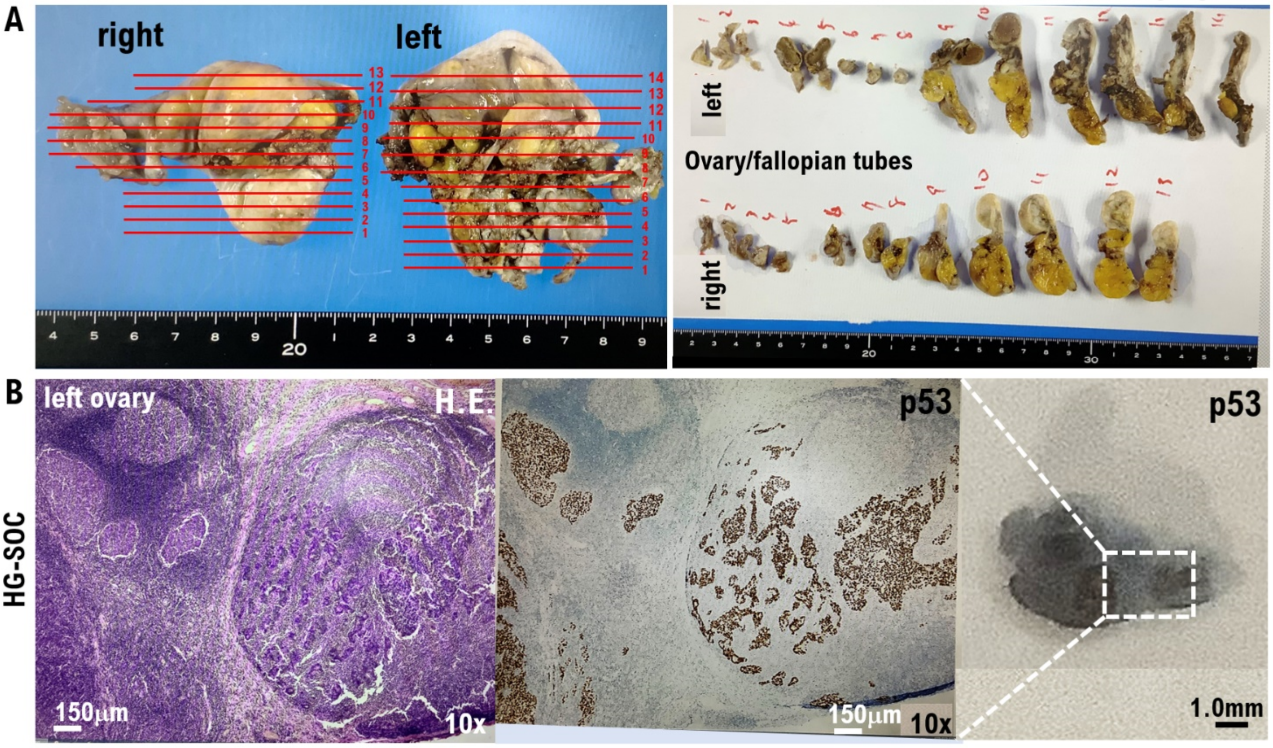

Figure S2. Detection of p53-positive cells in the left ovarian tissue derived from a patient with COVID-19. A. Cut surface of the excised fallopian tube and ovary: The mass found in the left ovary from a COVID-19 patient is a typical HG-SOC on macroscopic and microscopic examination. In the left ovary, a small grayish-white mass containing necrosis and hemorrhagic tissue is observed. Red lines and numbers indicate cross-sections in the gross findings of excised tissues. The numbers in red in the left panel of **A** match the numbers in red in the right panel of **A**. **B.** The left panel shows an H.E.-stained tissue section obtained from the left ovary of a patient with COVID-19. Recent research demonstrated that p53 is one of the biomarkers for HG-SOC. Therefore, a molecular pathological study was conducted, and the IHC study with antihuman p53 monoclonal antibody shows that p53-positive cells are observed in tumor-like mass in the left ovary (center panel and right panel). The results of this study confirmed the presence of an HG-SOC in the left ovary. Scale: 10×.

Supplementary Figure 3.

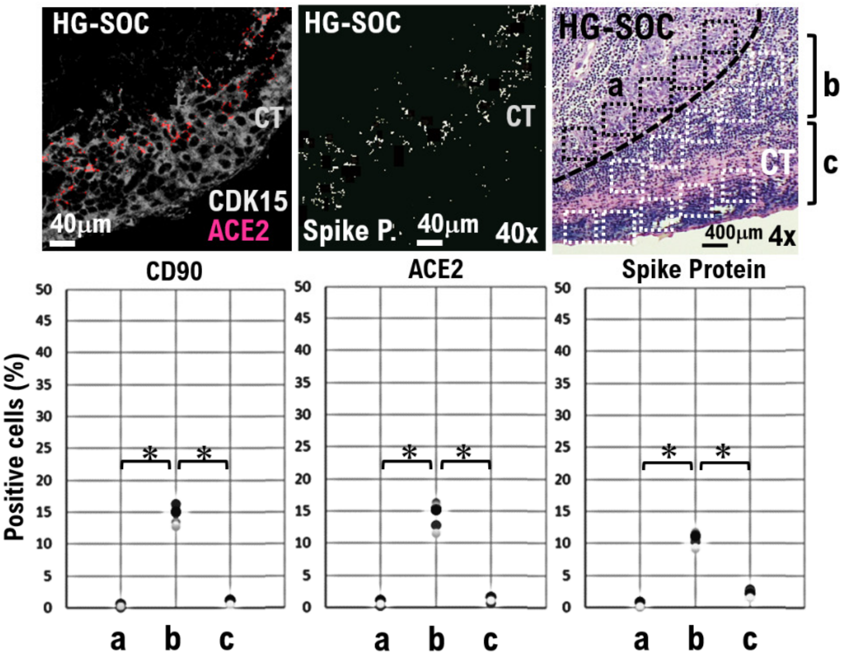

a: HG-SOC cells

b: some cells of the connecting tissue in contact with or in the vicinity of the micrometastatic niche of HG-SOC found in the ovary

c: cells of connecting tissue other than in the vicinity of micrometastatic niche of HG-SOC found in the ovary

Figure S3. Detection of SARS-CoV-2 spike glycoprotein (Spike P.) in some cells of the connecting tissue in contact with or in the micrometastatic niche of HG-SOC. IHC with anti-ACE2 monoclonal antibody or SARS-CoV-2 (Spike P.) antispike glycoprotein of monoclonal antibody is performed using ovarian tissues resected from the patient. The ACE2 expression is detected in some cells of the connecting tissue in contact with or in the micrometastatic niche of HG-SOC found in the patient's ovary. Spike glycoprotein of SARS-CoV-2 (Spike P.) is detected in some cells of the connecting tissue in contact with or in the micrometastatic niche of HG-SOC found in the ovary derived from the patient. CDK15 expression is detected in all cells of the connecting tissue in the ovary derived from the patient. The three types of bar graphs on the right show that CD90, ACE2, and spike glycoprotein (Spike P.) are clearly detected in some cells of the connecting tissue in contact with or in the micrometastatic niche of HG-SOC found in the ovary derived from the patient. The H.E. image shows the boundary between the HG-SOC tissue and the connecting tissue (CT) of the ovary. Comparing the expression in the **a** region and the **c** region, the expression status of CD90, ACE2, and Spike P. in the **b** region is statistically significantly higher (*$p < 0.001$).

Supplementary Figure 4.

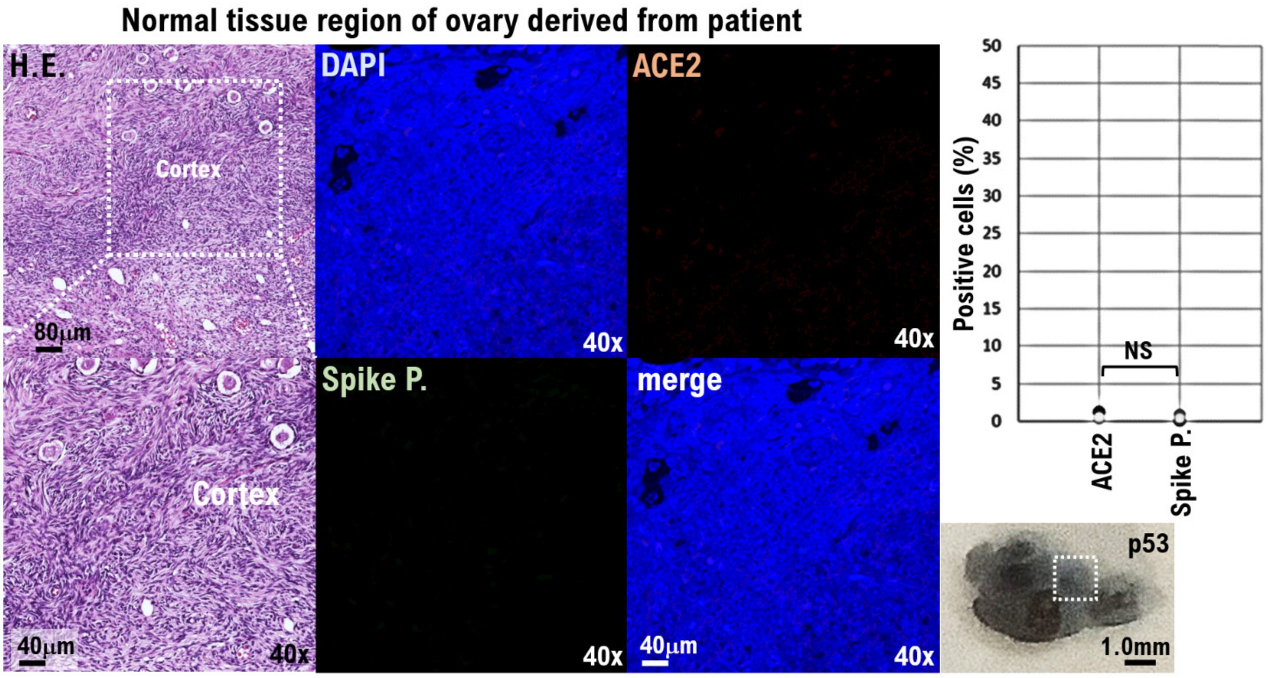

Figure S4. No ACE2 and spike glycoprotein of SARS-CoV-2 expression in the normal ovarian tissue. Particularly, in the normal tissue region of the ovary derived from the patient (H.E. images), IHC experiments show that the expression of ACE2 and spike glycoprotein of SARS-CoV-2 (Spike P.) is not clearly detected. The bar graph on the right shows that ACE2 and spike glycoprotein (Spike P.) are not expressed in the normal tissue region of the ovary derived from the patient. N.S., not significant; there is no statistically significant difference.

Supplementary Figure 5.

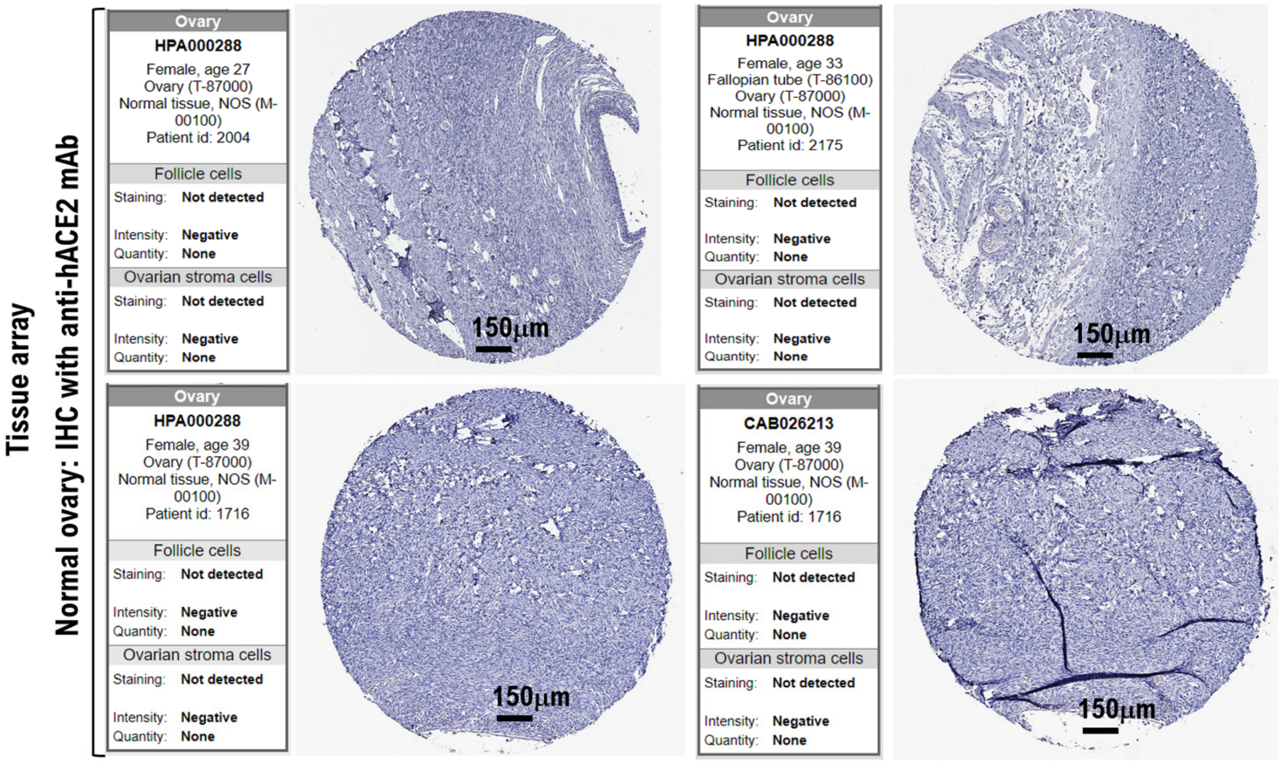

OVARY - Expression summary			
Protein expression Ovary[i]	n l m h		
	Follicle cells:	Not detected	
	Ovarian stroma cells:	Not detected	
RNA expression[i]	**Consensus:**	**1.8**	**nTPM**
	HPA:	0.8	nTPM
	GTEx:	1.8	nTPM
	FANTOM5:	3.2	Scaled Tags Per Million

The human protein atlas also demonstrated that the expressions of human ACE2 were not detected in follicle cells, stroma cells in healthy human ovary tissues.
https://www.proteinatlas.org/ENSG00000130234-ACE2/tissue/ovary

Figure S5. No ACE2 expression in normal ovarian tissues. IHC experiments with anti-human ACE2 monoclonal antibody do not provide the medical evidence that demonstrates ACE2 expression in any cells of the ovarian tissues, that is, follicle cells, and ovarian stroma cells using a normal ovarian tissue array.

Supplementary Figure 6.

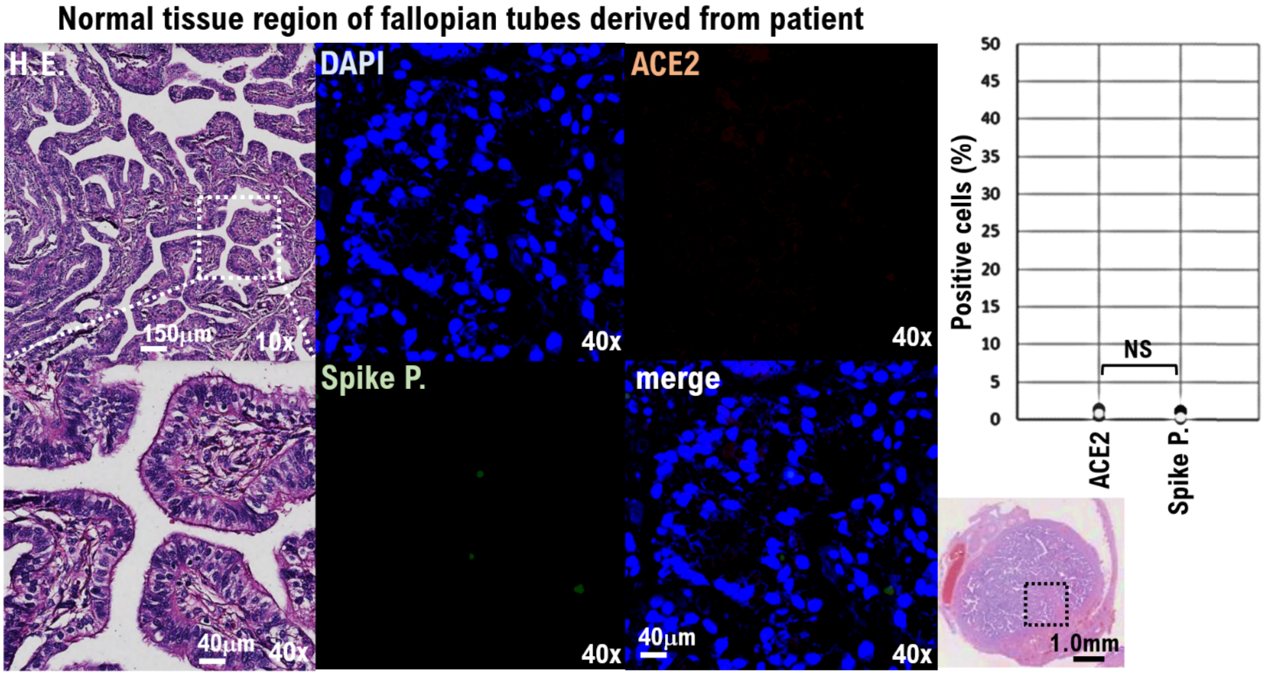

Figure S6. No expression of ACE2 and SARS-CoV-2 spike glycoprotein in the normal tissue region of the fallopian tubes derived from the patient. Particularly, in the normal tissue region of fallopian tubes derived from the patient (H.E. images), IHC experiments show that the expression of ACE2 and spike glycoprotein of SARS-CoV-2 (Spike P.) is not clearly detected. The bar graph on the right shows that ACE2 and spike glycoprotein (Spike P.) are not expressed in the normal tissue region of the fallopian tubes derived from the patient. N.S., not significant; there is no statistically significant difference.

Supplementary Figure 7.

Tissue array

Normal fallopian tubes: IHC with anti-hACE2 mAb

Ciliated cells (cell body)	Not detected	Not detected	Not detected	Not detected
Ciliated cells (cilia axoneme)	Not detected	Not detected	Not detected	Not detected
Ciliated cells (ciliary rootlets)	Low	Low	Low	Low
Ciliated cells (tip of cilia)	Not detected	Not detected	Not detected	Not detected
Glandular cells	-	-	-	-
Non-ciliated cells	Not detected	Not detected	Not detected	Not detected

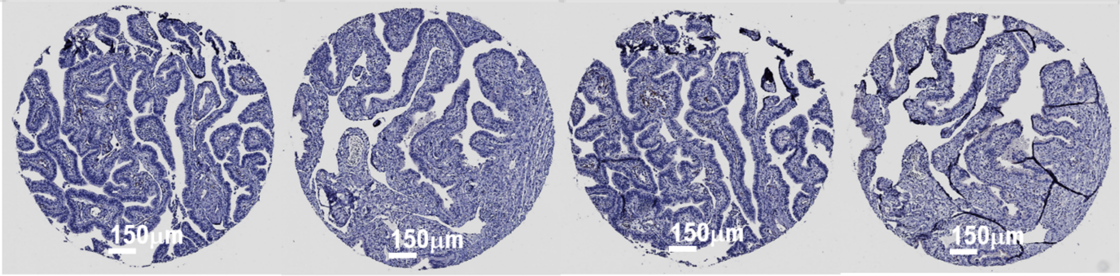

Figure S7. No expression of ACE2 in the normal fallopian tube tissues. IHC experiments with anti-human ACE2 monoclonal antibody do not provide the medical evidence that demonstrates ACE2 expressions in any cells of the fallopian tube tissues, *that is*, ciliated cells (cell body), ciliated cells (cilia axoneme), ciliated cells (ciliary rootlets), ciliated cells (tip of cilia), glandular cells, and nonciliated cells using normal fallopian tube tissue array.

Supplementary Figure 8.

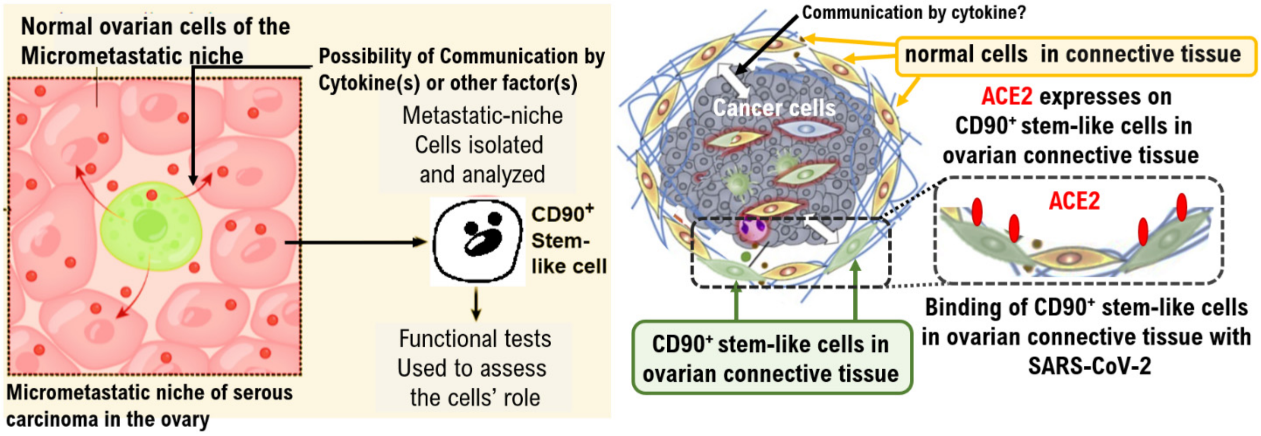

Figure S8. The mechanism of changes in the molecular pathology attributed to the SARS-CoV-2 infection. In the micrometastatic niche, epithelial cells *etc*. that are in contact with cancer cells are differentiated into stem-like cells and progenitor cells by some cytokine(s) or other factor(s). ACE2, the receptor for SARS-CoV-2 markedly expresses in CD90-positive stem-like cells or CD90-positive progenitor cells that are in contact with metastatic tumors in organs and tissues that did not express ACE2. Therefore, for COVID-19 patients with metastasis to other organs, SARS-CoV-2 infection is observed in cells that are in contact with metastatic tumors in the micrometastatic niche.

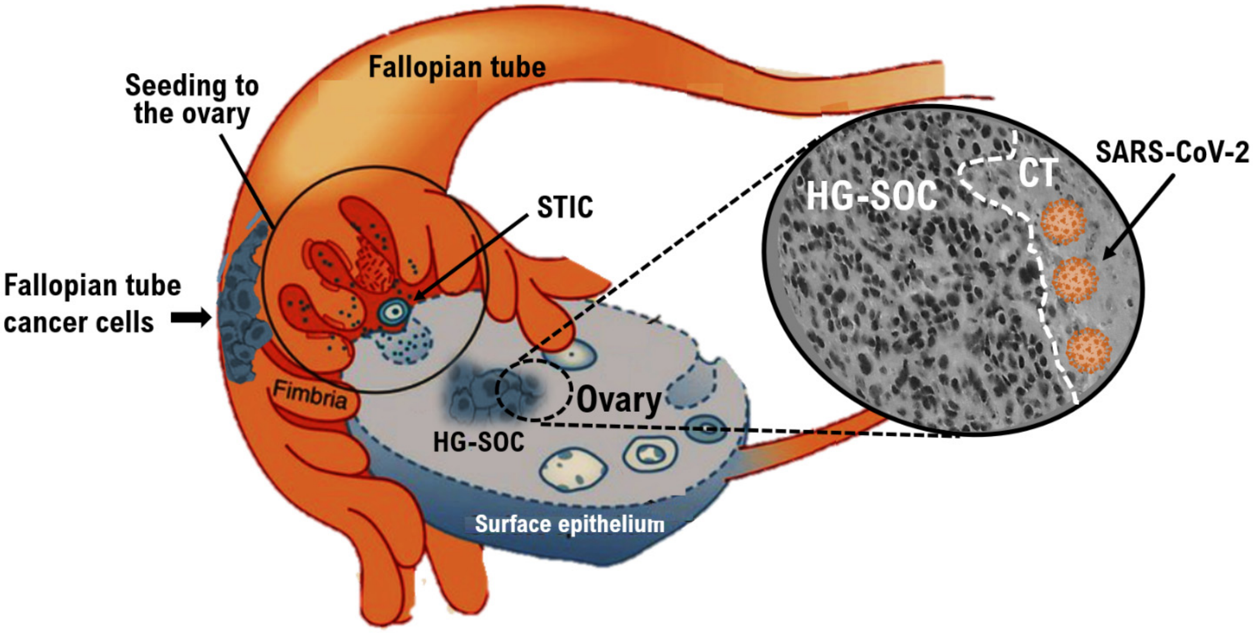

Figure S9. SARS-CoV-2 infection in ovarian histiocytes in contact with the micrometastatic niche of HG-SOC. The origin of HG-SOC is STIC and/or epithelial malignancies in the fallopian tubes. STIC is most likely the precursor lesion of high-grade serous pelvic carcinomas, carcinosarcoma, and undifferentiated carcinoma. There is a window of 10 to 20 years between the development of abnormal cells, or lesions, in the fallopian tubes and the initiation of ovarian cancer. In the process of forming the micrometastatic niche in multiple organs, epithelial cells and stromal cells in contact with CTCs are initialized by secretory factors from CTCs that comprise the micrometastatic niche. ACE2 was possibly expressed in the epithelial stem-like cells or progenitor cells. ACE2 expression is observed, and SARS-CoV-2 particles are detected in ovarian histiocytes in contact with the micrometastatic niche of HG-SOC. It is considered that the factors secreted from the micrometastatic niche of HG-SOC may induce SARS-CoV-2 infection and proliferation. ACE2, angiotensin-converting enzyme 2; CTC, circulation tumor cell; HG-SOC, high-grade serous ovarian cancer; SARS-CoV-2, severe acute respiratory syndrome coronavirus 2; STIC, serous tubal intraepithelial carcinoma.

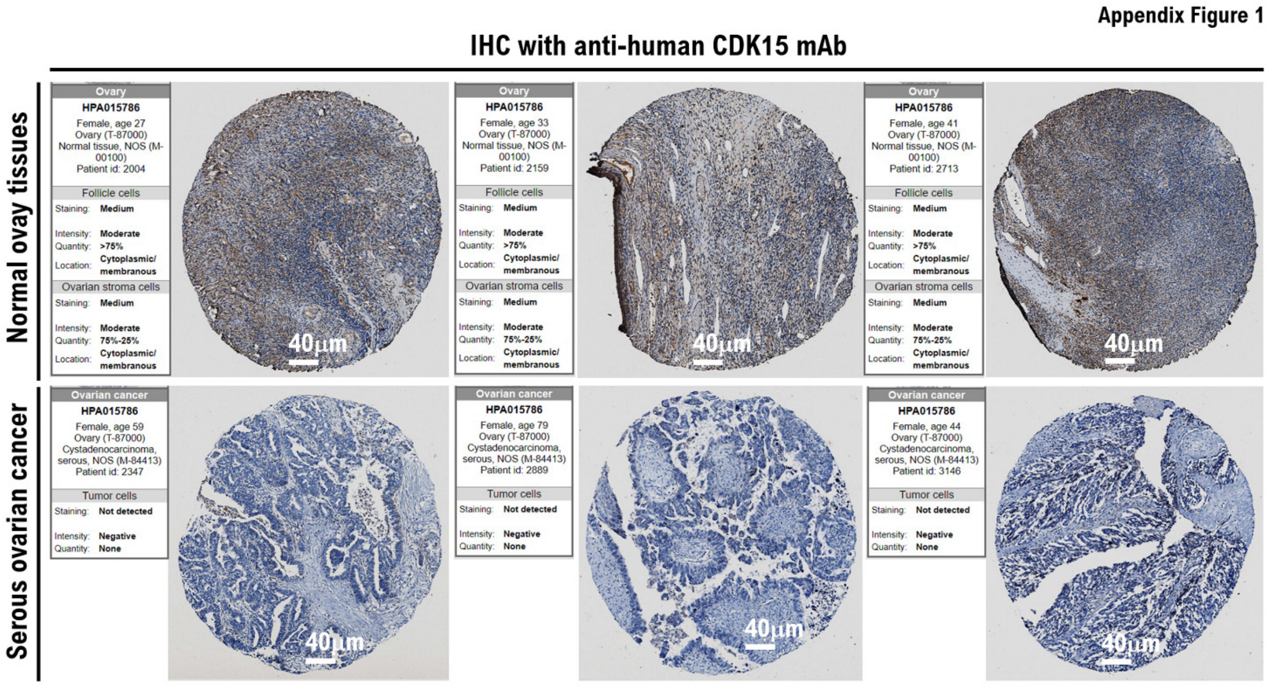

Figure S10. The expression of CDK15 in connective tissue/stroma cells; no expression of CDK15 in serous ovarian cancer. IHC experiments with anti-human CDK15 monoclonal antibody provide the medical evidence that demonstrates CDK15 expressions in connective tissue/stroma cells in ovarian tissues (upper panels). From the IHC experiment results with antihuman CDK15 monoclonal antibody, the expression of CDK15 was not clearly detected in serous ovarian cancer (lower panels).
