# Supplementary material for: Pathological Evidence for Residual SARS-CoV-2 in the Micrometastatic Niche of a Patient with Ovarian Cancer
Source: Curr Issues Mol Biol. 2022 Nov 26;44(12):5879–89. doi: 10.3390/cimb44120400 (PMC9776863; doi:10.3390/cimb44120400)
Supplement: Supplementary file 1 [file cimb-44-00400-s001.zip › Form.09.08.2022.pdf]

Copyrights

Copyright and Licensing

For all articles published in MDPI journals, copyright is retained by the authors. Articles are licensed under an open access Creative Commons CC BY 4.0 license, meaning that anyone may download and read the paper for free. In addition, the article may be reused and quoted provided that the original published version is cited. These conditions allow for maximum use and exposure of the work, while ensuring that the authors receive proper credit.

In exceptional circumstances articles may be licensed differently. If you have specific condition (such as one linked to funding) that does not allow this license, please mention this to the editorial office of the journal at submission. Exceptions will be granted at the discretion of the publisher.

Reproducing Published Material from other Publishers

It is absolutely essential that authors obtain permission to reproduce any published material (figures, schemes, tables or any extract of a text) which does not fall into the public domain, or for which they do not hold the copyright. Permission should be requested by the authors from the copyright holder (usually the Publisher, please refer to the imprint of the individual publications to identify the copyright holder).

Permission **is required** for:

1. Your own works published by other Publishers and for which you did not retain copyright.
2. Substantial extracts from anyone's works or a series of works.
3. Use of Tables, Graphs, Charts, Schemes and Artworks if they are unaltered or slightly modified.
4. Photographs for which you do not hold copyright.

Permission **is not required** for:

1. Reconstruction of your *own* table with data already published elsewhere. Please notice that in this case you must cite the source of the data in the form of either "Data from..." or "Adapted from...".
2. Reasonably short quotes are considered *fair use* and therefore do not require permission.
3. Graphs, Charts, Schemes and Artworks that are completely redrawn by the authors and significantly changed beyond recognition do not require permission.

Obtaining Permission

In order to avoid unnecessary delays in the publication process, you should start obtaining permissions as early as possible. If in any doubt about the copyright, apply for permission. MDPI cannot publish material from other publications without permission.

The copyright holder may give you instructions on the form of acknowledgement to be followed; otherwise follow the style: "Reproduced with permission from [author], [book/journal title]; published by [publisher], [year].' at the end of the caption of the Table, Figure or Scheme.

September 08, 2022
Takuma Hayashi
National Hospital Organization Kyoto Medical Center
