# Supplementary material for: Pathological Evidence for Residual SARS-CoV-2 in the Micrometastatic Niche of a Patient with Ovarian Cancer
Source: Curr Issues Mol Biol. 2022 Nov 26;44(12):5879–89. doi: 10.3390/cimb44120400 (PMC9776863; doi:10.3390/cimb44120400)
Supplement: Supplementary file 1 [file cimb-44-00400-s001.zip › Institutional Review Board Statement.pdf]

Institutional Review Board Statement and Consent to Participate.

These experiments with human tumor tissues derived from patients with high grade-serous ovarian cancer were conducted at Shinshu University and National Hospital Organization Kyoto Medical Center in accordance with institutional guidelines (i.e., IRB approval no. M192, H31-cancer-2). The authors attended research ethics education through the Education for Research Ethics and Integrity (APRIN e-learning program (eAPRIN)). The completion numbers for the authors are AP0000151756, AP0000151757, AP0000151769, and AP000351128. Consent to participate was required as this research was considered clinical research. Subjects signed an informed consent form when they were briefed on the clinical study and agreed with content of the research.

Ethic Committee Name: Institutional Review Board (IRB) of Shinshu University
Approval Code: M192
Approval Date: April 05, 2014, and June 16, 2016

Ethic Committee Name: Institutional Review Board (IRB) of National Hospital Organization Headquarter
Approval Code: H31-cancer-2
Approval Date: November 09, 2019, and June 17, 2022
